# Molecular targeting of renal cell carcinoma by an oral combination

**DOI:** 10.1038/s41389-020-0233-0

**Published:** 2020-05-19

**Authors:** Andre R. Jordan, Jiaojiao Wang, Travis J. Yates, Sarrah L. Hasanali, Soum D. Lokeshwar, Daley S. Morera, Nagarajarao Shamaladevi, Charles S. Li, Zachary Klaassen, Martha K. Terris, Muthusamy Thangaraju, Amar B. Singh, Mark S. Soloway, Vinata B. Lokeshwar

**Affiliations:** 1 Department of Biochemistry and Molecular Biology, Medical College of Georgia, Augusta University, 1410 Laney Walker Blvd., Augusta, GA 30912 USA; 2 Sheila and David Fuente Graduate Program in Cancer Biology, University of Miami-Miller School of Medicine, Miami, 1600 NW 10th Avenue, Miami, FL 33136 USA; 3 Honors Program in Medical Education, University of Miami-Miller School of Medicine, Miami, 1600 NW 10th Avenue, Miami, FL 33136 USA; 4 GeneChem Diagnostics Laboratory, Miami, FL 33157 USA; 5 Department of Surgery, Division of Urology, Medical College of Georgia, Augusta University, 1410 Laney Walker Blvd., Augusta, GA 30912 USA; 6 Department of Biochemistry and Molecular Biology, University of Nebraska Medical Center, Omaha, NE, USA; 7 Memorial Healthcare System, Aventura, FL 33180 USA; 8 Present Address: Travis Yates: QualTek Molecular Laboratories, King of Prussia, PA USA

**Keywords:** Renal cell carcinoma, Target identification, Renal cell carcinoma, Target identification, Renal cell carcinoma

## Abstract

The 5-year survival rate of patients with metastatic renal cell carcinoma (mRCC) is <12% due to treatment failure. Therapeutic strategies that overcome resistance to modestly effective drugs for mRCC, such as sorafenib (SF), could improve outcome in mRCC patients. SF is terminally biotransformed by UDP-glucuronosyltransferase-1A9 (A9) mediated glucuronidation, which inactivates SF. In a clinical-cohort and the TCGA-dataset, A9 transcript and/or protein levels were highly elevated in RCC specimens and predicted metastasis and overall-survival. This suggested that elevated A9 levels even in primary tumors of patients who eventually develop mRCC could be a mechanism for SF failure. 4-methylumbelliferone (MU), a choleretic and antispasmodic drug, downregulated A9 and inhibited SF-glucuronidation in RCC cells. Low-dose SF and MU combinations inhibited growth, motility, invasion and downregulated an invasive signature in RCC cells, patient-derived tumor explants and/or endothelial-RCC cell co-cultures; however, both agents individually were ineffective. A9 overexpression made RCC cells resistant to the combination, while its downregulation sensitized them to SF treatment alone. The combination inhibited kidney tumor growth, angiogenesis and distant metastasis, with no detectable toxicity; A9-overexpressing tumors were resistant to treatment. With effective primary tumor control and abrogation of metastasis in preclinical models, the low-dose SF and MU combinations could be an effective treatment option for mRCC patients. Broadly, our study highlights how targeting specific mechanisms that cause the failure of “old” modestly effective FDA-approved drugs could improve treatment response with minimal alteration in toxicity profile.

## Introduction

The 5-year survival rate of patients with metastatic renal cell carcinoma (mRCC) is <12%^1–3^. Approximately 30% of patients have metastasis at initial diagnosis and another ~30% develop metastasis, even after surgical intervention. Tyrosine kinase and mTOR inhibitors are approved as first- or second-line treatments for mRCC^4,5^. Recently, immune checkpoint inhibitors were approved as a first-line treatment for treatment-naïve patients with intermediate or high-risk advanced RCC^6,7^. However, the majority of patients experience tumor progression due to treatment resistance^8–10^.

Sorafenib (SF) is a multi-kinase inhibitor with anti-angiogenic and anti-proliferative properties that was FDA-approved for the treatment of mRCC^3,11^. However, due to its modest efficacy and treatment resistance, it is generally used only if other therapies have failed^12–14^. Studies have implicated EGFR, c-Jun, PI3K/AKT, and Ras/Raf/MEK/ERK pathways, as well as, autophagy and/or epithelial-mesenchymal transition (EMT) in SF failure in the clinic^15,16^. However, no studies have identified mechanisms directly relating to SF activity, nor inter-patient differences accounting for variable response to SF.

4-methylumbelliferone (MU; 7-hydroxy-4-methylcoumerin or Hymecromone) has choleretic and antispasmodic properties, but it lacks the anti-sperminogenic and anti-aromatase activities of coumarin, as well as, the anticoagulant activity of coumadin^17–22^. The maximum tolerated dose of MU in mice is 2.8–7.3 g/kg (NIOS registry). We and others have shown that inhibition of HA synthesis is the major mechanism of the anticancer properties of MU as a single agent (IC_50_ = 0.4 mM)^20,22–25^. However, in RCC models, we showed that at plasma achievable levels (~5 µM) SF synergized with MU, and this combination (SF + MU) had potent antitumor efficacy at doses where both agents individually were ineffective^26–29^. In the synergistic combination, MU doses were also 2–4-fold less than its IC_50_ for inhibiting HA synthesis^26^. Therefore, the mechanism by which low doses of MU improve the efficacy of SF is independent of the inhibition of HA signaling that occurs at higher doses^20,22–25^.

SF is metabolized primarily in the liver; oxidative metabolism by Cytochrome P450 3A4 (CYP3A4) is the major pathway yielding SF N-oxide metabolites. CYP3A4 is also expressed in the normal kidney in proximal tubular epithelial cells and in tumor cells in RCC^30,31^. As a minor pathway, in the liver SF also directly undergoes terminal biotransformation by UDP-glucuronosyltransferase -1A9 (UGT1A9 or A9); A9 is also the major isoform in the kidney^32–35^. Glucuronidation is usually the terminal biotransformation and 15–19% of the SF dose is excreted in urine as a glucuronide metabolite^32,35^.

We evaluated if MU alters SF metabolism and improves its efficacy. Efficacy of SF + MU combination were evaluated in preclinical models of RCC and endothelial cells, including a SF-resistant spontaneously metastatic model. Our study shows that A9 is overexpressed in RCC cells. MU alone and the combination downregulate A9 expression and inhibit SF glucuronidation. Tumors from patients who develop mRCC overexpress A9 transcript and protein. A9 expression correlates with clinical outcomes. Patient-derived tumorspheroids and tumor models reveal that by downregulating A9, MU improves the antitumor and antimetastatic activities of SF. Therefore, SF + MU combination could be a potential treatment for mRCC.

## Results

### Identification of A9 as a possible target for SF + MU combination

**Fig. 1:**
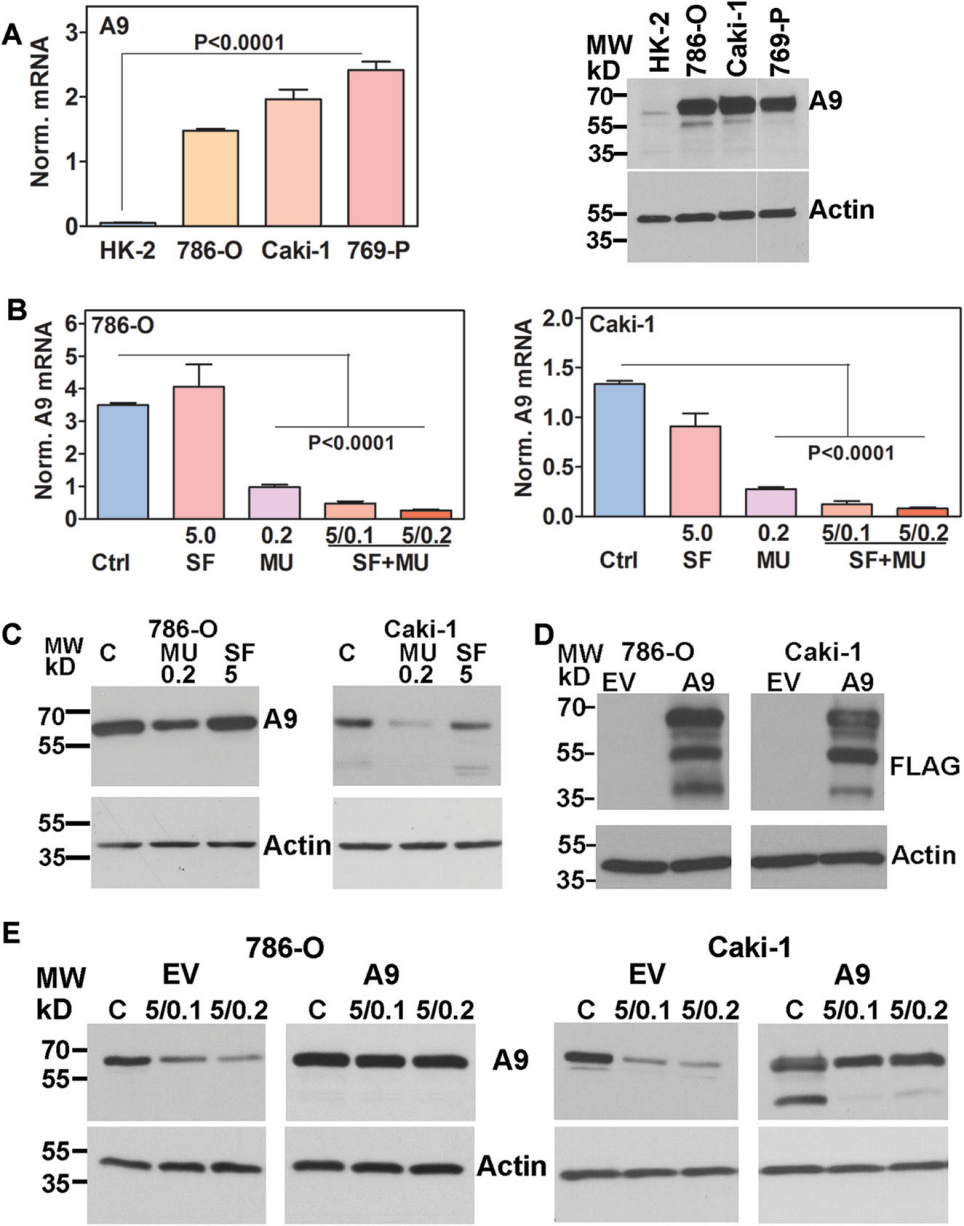
Analysis of A9 expression in RCC cells. **a** Basal level A9 mRNA and protein expression in normal kidney (HK-2) and RCC cell lines. Normalized mRNA levels: Mean ± SD (*n* = 3). For the immunoblot, all samples were analyzed on the same gel with the same exposure time; a gap denotes the lanes that were not contiguous in the same gel/blot. **b** A9 mRNA expression in 786-O and Caki-1 cells either untreated (Ctrl) or treated with SF, MU, or SF (5 μM) + MU (0.1 or 0.2 mM). Data: Mean ± SD (*n* = 3). **c** Immunoblot analysis of A9 expression in 786-O and Caki-1 cells untreated (C: control) or treated with MU (0.2 mM), SF (5 μM). **d** Immunoblot analysis for FLAG tag in EV and A9 transfectants. **e** Immunoblot analysis of A9 expression in EV and A9 transfectants treated or untreated with SF + MU (5/0.1, 5/0.2). **a**, **c**–**e** Loading control in immunoblots: actin.

We previously demonstrated that the combination of SF with MU had synergistic efficacy against RCC cells both in vitro and in a subcutaneous xenograft^26^. Optimal synergy between SF and MU was observed at concentrations (5 µM SF; 0.1–0.2 mM MU) where both agents individually were ineffective^26^. The combinations (SF + MU: 5/0.1, 5/0.2) were equally effective in both VHL+ and VHL− RCC cell lines; VHL is a tumor suppressor that is frequently mutated or deleted in RCC^36^. Since both CYP3A4 and A9 metabolize SF and are expressed in the kidney^31–33,35,37,38^, we investigated if their expression, and consequently, SF metabolism are altered in RCC cells treated with the SF and MU combination (SF + MU). While A9 transcript and protein levels were about 15-fold elevated in RCC cell lines, CYP3A4 expression was similar in the normal kidney epithelial line (HK-2) and RCC cells (Fig. 1a; Supplementary data: Fig. 1A, Table 1). Moreover, MU treatment alone downregulated A9 transcript and protein expression by 3 to 4-fold in 786-O and Caki-1 cells (Fig. 1b, c). These cell lines were chosen as, Caki-1 is VHL+ and 786-O cell line is VHL−^39^. The SF + MU combination was similarly effective in downregulating A9 transcript expression (Fig. 1b). However, CYP3A4 expression was not affected by either MU or SF + MU treatments (Supplementary Fig. 1B). To study if downregulation of A9 by SF + MU treatment contributed to the observed antitumor effects of the treatment, we stably expressed FLAG-tagged A9 protein in 786-O and Caki-1 cells (Fig. 1d).

As shown in Fig. 1e, SF + MU treatment downregulated A9 protein levels in the EV-transfectants. However, in A9-transfectants, which express A9 under a viral promoter, A9 levels remained largely unaffected by SF + MU treatment. A9 expression in the A9 transfectants was comparable to that found in RCC tissues (described below). Reverse HPLC analysis of SF or SF + MU treated 786-O EV cells showed inhibition of SF glucuronidation by the treatment, whereas SF glucuronidation was only partially inhibited by the treatment in A9 transfectants (Supplementary Fig. 1C).

### A9-expression is increased in RCC specimens and associates with clinical outcome and SF-resistance

**Fig. 2:**
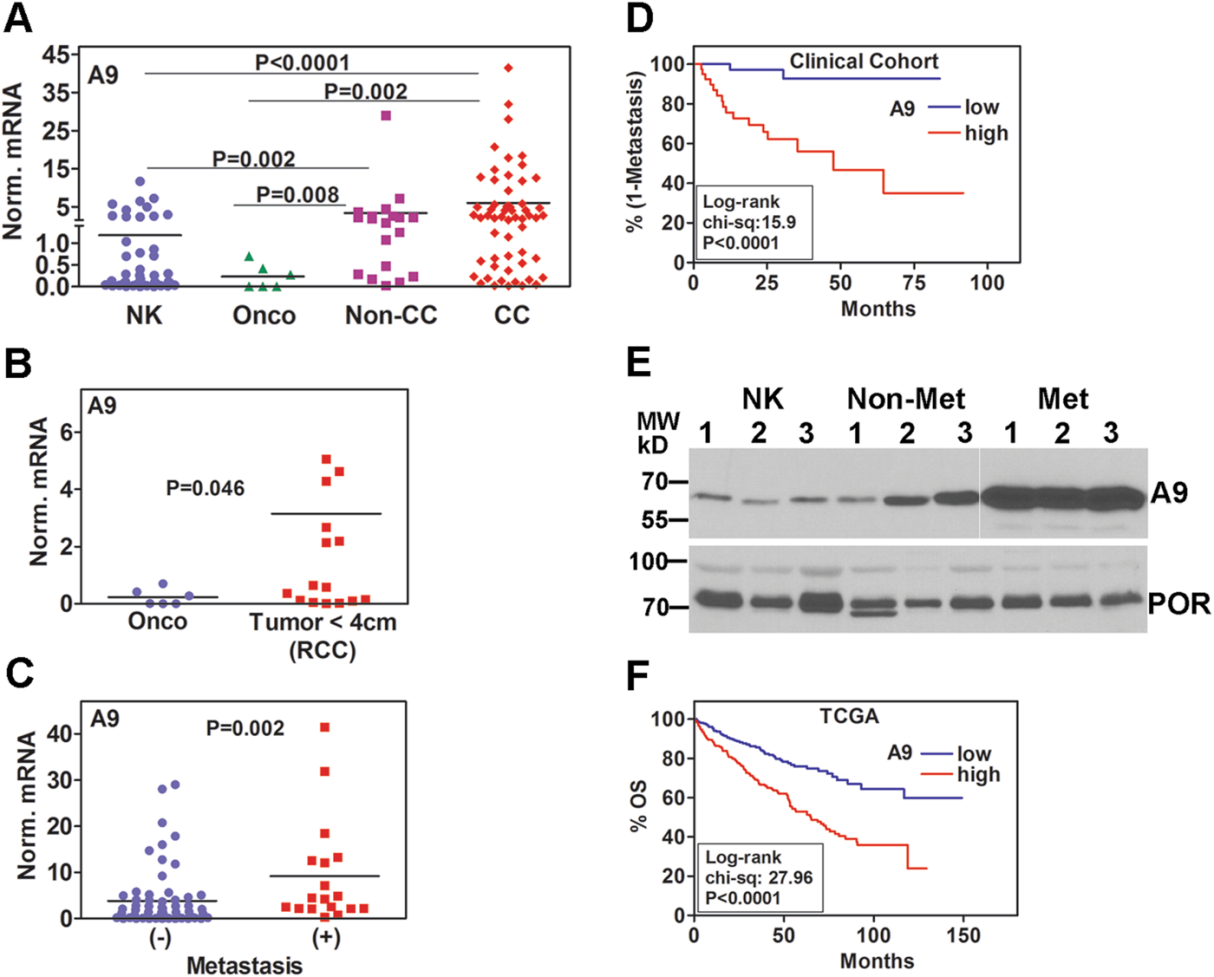
Analysis of A9 expression in RCC specimens. **a–c** Normalized A9 mRNA levels in normal kidney (NK) and RCC tissues. **a** A9 mRNA levels in NK (*n* = 51), oncocytoma (Onco; *n* = 6), non-clear cell RCC (non-CC RCC: Chromophobe [*n* = 5], papillary [*n* = 10], sarcomatoid [*n* = 2], collecting duct [*n* = 2]), and clear cell RCC (CC; *n* = 58). **b** Comparison of A9 levels between oncocytoma (size, 4.0 ± 1.0 cm; *n* = 6) and small RCC tumors (<4 cm; *n* = 17). **c** A9 levels in RCC specimens from patients who did not (*n* = 64) or developed (*n* = 18) metastasis during follow-up. **a**–**c** Data: mean ± SEM. **d** Kaplan–Meier plot showing risk-stratification of the cohort by A9 mRNA levels for metastasis (*n* = 83). **e** Immunoblot analysis of A9 in the microsomes prepared from NK (*n* = 3) and tumor specimens from patients who either did not (Non-Met; *n* = 3) or developed metastasis during follow-up (Met; *n* = 3). Microsomes were prepared from fresh-frozen tissues by homogenizing them in eight times the volume of Hepes-sucrose buffer (20 mM Hepes, 154 mM KCl, 250 mM sucrose, 2 mM MgCl_2_ pH 7.2, with protease cocktail) and sequentially centrifugation at 1500×*g* for 10 min, 8000×*g* for 20 min, and 100,000×*g* for 70 min twice. Microsomes were stored in 20 mM Tris-HCl, 250 mM sucrose, pH 7.5, buffer. Microsome marker, cytochrome P450 reductase was used for the normalization of A9 expression in the microsomes. Note: all samples were analyzed on the same gel with the same exposure time; a gap denotes the lanes that were not contiguous in the same gel/blot. **f** Kaplan–Meier plot showing risk-stratification of the TCGA dataset for OS (*n* = 542).

Since RCC cells showed increased A9 levels when compared to HK-2 cells, we measured A9 expression in tumor and NK tissues from RCC patients (clinical-cohort; Supplementary Table 2). The majority of tumor specimens in the cohort were of clear cell (cc) RCC (58/83). Compared to NK specimens, A9 mRNA levels were about 6-fold upregulated in ccRCC tumors; the increase in non-ccRCC tumors (papillary, chromophobe, collecting duct, and sarcomatoid), was ~3-fold (Fig. 2a). The increase in A9 levels in both ccRCC and non-ccRCC tumors was also significantly higher when compared to oncocytoma (Fig. 2a). A9 levels were ~16-fold higher in small RCC tumors (<4 cm) compared to oncocytoma (Fig. 2b). Moreover, A9 mRNA levels were 2.6-fold higher in tumors from patients who either had or developed metastasis during follow-up, than in tumors from patients who did not (Fig. 2c). In both univariate and multivariate analyses, A9 levels were significant predictors of metastasis (Supplementary Tables 3, 4). Furthermore, Kaplan–Meier plots showed that high A9 levels significantly stratified patients into higher risk for metastasis (Fig. 2d). In renal cells, A9 is localized within the endoplasmic reticulum^34^. Therefore, we analyzed A9 protein levels in microsome preparations from NK, and kidney tumors from patients in the clinical cohort. A9 levels were 5–10-fold elevated in tumor microsomes and the increase was higher in tumors from patients who developed metastasis (Fig. 2e).

We next analyzed whether A9 expression correlated with clinical outcome in a ccRCC TCGA-cohort of 542 patients (Supplementary Table 2). Although clinical outcome in terms of OS but not metastasis are available. Nevertheless, A9 levels significantly correlated with M-stage (*P* = 0.0015; odds ratio = 2.08; 95% CI: 1.3–3.3). In univariate analysis, A9 levels associated with OS and in multivariate analysis age, M-stage, and A9 levels were independent predictors of metastasis (Supplementary Tables 3, 4). Kaplan–Meier plots showed that A9 levels significantly stratified patients for risk for death (Fig. 2f).

### A9-overexpression attenuates cytotoxicity of SF + MU

**Fig. 3:**
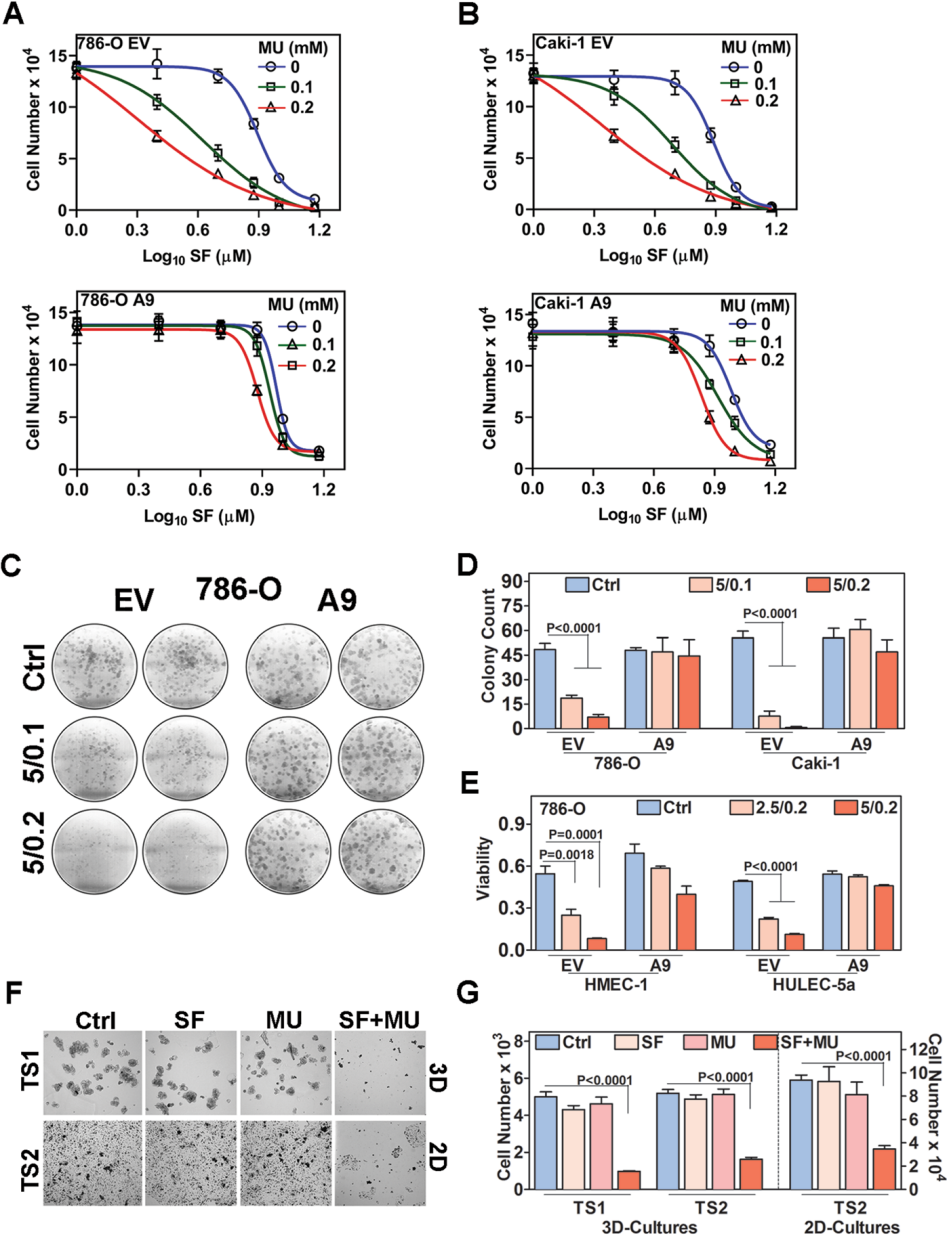
Effect of SF, MU, or SF + MU treatment on cell growth. **a**–**d** EV and A9 transfectants of 786-O (**a**, **c**, **d**) and Caki-1 (**b**, **d**) cells were treated with SF, MU, and SF + MU at indicated concentrations. Viable cell counts at 72-h are shown (**a**, **b**). The line graphs indicate non-linear regression sigmoidal curves generated using variable slope equation; symbols are actual data points. Photograph (**c**) and quantification (**d**) of colonies on day 7. Ctrl: control; SF + MU treatment: 5/0.1 and 5/0.2. **e** Viability of endothelial cells (HMEC-1; HULEC-5a) in co-cultures with 786-O transfectants, following SF + MU treatment as measured by MTT-assay. Viability data are O.D. (575 nm) – O.D. (650 nm – reference). **f**, **g** Patient-derived tumorspheres (TS1, TS2) or 2D-primary cultures of tumor cells (TS2) were treated with SF (5 µM), MU (0.2 mM), or SF + MU (5/0.2) for 48-h. Tumorspheres and 2D cultures were photographed at 100X magnification (**f**). Viability of tumorspheres and 2D-cultures was determined by cell counting (**g**) Data in **a**, **b**, **d**, **e**, **g** mean ± SD (*n* = 3 to *n* = 4).

Since A9 expression was elevated in RCC cells and tumors and downregulated by MU, we examined the sensitivity of EV and A9 transfectants to SF in combination with different doses of MU. In a dose-dependent manner, SF alone inhibited proliferation of 786-O and Caki-1 EV transfectants with an IC_50_ of ~7.8 µM (Fig. 3a, b; Supplementary Table 5). Combination of SF with MU at 0.1 or 0.2 mM dose lowered the IC_50_ by 1.9- to 3.9-fold in 786-O and 1.6- to 3.5-fold in Caki-1 EV cells, respectively. A9 transfectants were slightly resistant to SF and the SF + MU combinations could lower the IC_50_ by only 24% to 40% at 0.2 mM dose (Fig. 3a, b; Supplementary Table 5).

To examine whether downregulation of A9 would sensitize the RCC cells to SF treatment, we generated A9 shRNA transfectants of both 786-O and Caki-1 cells using two different shRNA constructs. In these transfectants, A9 expression was downregulated by ≥80% (Supplementary Fig. 2A, Supplementary Table 1). When compared to the control shRNA transfectants, IC_50_ for growth inhibition by SF alone was 2.5–3.1-fold lower in the A9 shRNA transfectants (Supplementary Fig. 2B, C; Supplementary Table 5).

At the 400 b.i.d oral dose, the plasma level of SF is ~5 µM^26–29^. To determine if MU at 0.1 and 0.2 mM doses can improve the response to SF at the pharmacologically achievable dose of 5 µM, we performed subsequent experiments using 5 µM SF + 0.1 mM MU (5/0.1) and 5 µM SF + 0.2 mM MU (5/0.2) combinations. At these doses, SF + MU inhibited clonogenic survival by 86–98.8% in EV-transfectants, but A9-transfectants were resistant (Fig. 3c, d). In A9-shRNA transfectants, SF alone inhibited clonogenic survival by >90% (Supplementary Fig. 2D).

RCC is known for its pro-angiogenic environment in which tumor cells stimulate growth and motility of endothelial cells^39^. Therefore, we assessed the effect of SF + MU on the viability of HMEC-1 and HULEC-5a microvessel endothelial cells, co-cultured with EV- and A9-transfectants of both 786-O and Caki-1 cells. When co-cultured with EV-transfectants, both HMEC-1 and HULEC-5a remained sensitive to SF + MU; 84.8% reduction in viable cells at 5/0.2 dose. However, endothelial cells were resistant to treatment when co-cultured with A9-transfectants (Fig. 3e, Supplementary Fig. 2E).

We also evaluated the therapeutic potential of the SF + MU combination on two patient-derived RCC tumorspheres (TS1; TS2). SF + MU (5/0.2) inhibited the anchorage independent growth (3D-culture) of TS1 and TS2 by 69–81%, while SF and MU alone were ineffective (Fig. 3f, g). Similarly, SF + MU caused >60% inhibition of TS2 growth in 2D-cultures (Fig. 3f, g).

### A9 expression attenuates cell cycle arrest and apoptosis induction by SF + MU

**Fig. 4:**
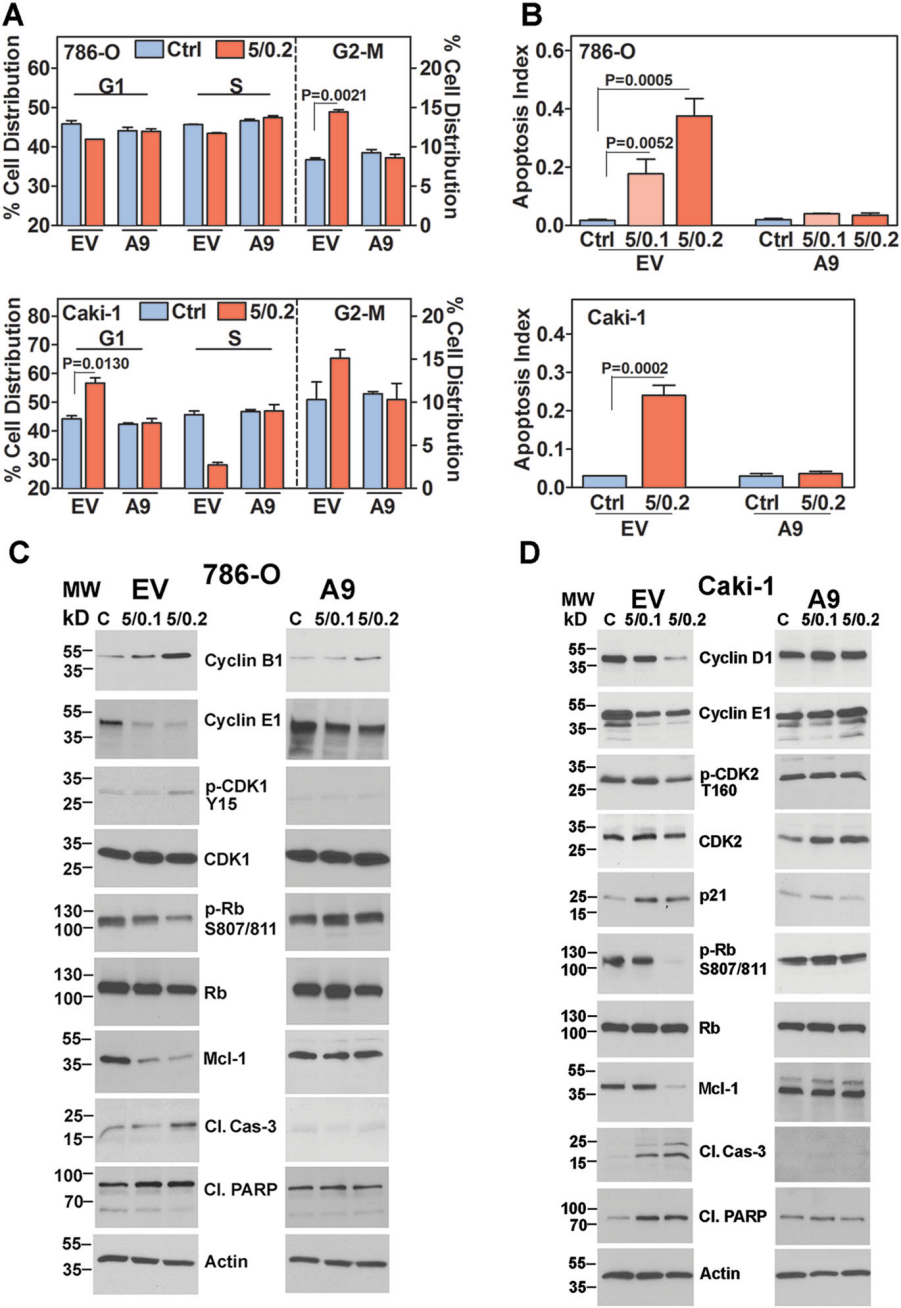
Effect of SF + MU treatment on cell cycle arrest and apoptosis. EV and A9 transfectants were treated with SF + MU (5/0.1, 5/0.2). **a** Cell cycle analysis after 24-h treatment. **b** Apoptosis measured following 48-h treatment. Data in A and B: Mean ± SD (*n* = 3). **c**, **d** Immunoblot analyses for cell cycle and apoptosis indicators after SF + MU treatment. Loading control: actin.

To further evaluate the mechanism of SF + MU-mediated inhibition of cell growth, we conducted cell cycle analysis on SF + MU treated EV- and A9-transfectants. Within 24-h of treatment, SF + MU mainly caused G2-M arrest in 786-O EV-transfectants. At 5/0.2 SF + MU dose, 1.74-fold more cells were in the G2-M phase. In Caki-1 EV-transfectants, SF + MU mainly induced cell cycle arrest in the G0-G1 phase, with a corresponding 1.6-fold decrease in the S-phase (Fig. 4a). However, SF + MU failed to induce cell cycle arrest in A9-transfectants in both cell types. G2-M-arrest in 786-O EV-transfectants was validated by decreased p-Rb and cyclin E1 levels, and increased Cyclin B1 and p-CDK1 levels (Fig. 4c). Similarly, decreased levels of p-Rb, Cyclin E1, Cyclin D1, and p-CDK2, and increased p21 levels, validated G0-G1 arrest in Caki-1 EV-transfectants by SF + MU (Fig. 4d). Contrarily, SF + MU had little to no effect on the expression of cell cycle markers in A9-transfectants.

Since SF + MU treatment caused cell cycle arrest in EV-, but not A9-transfectants, we further determined whether the combination could induce apoptosis in these transfectants. After 48-h of treatment SF + MU induced apoptosis by ≥8-fold in EV-transfectants, compared to untreated control (Fig. 4b). However, SF + MU failed to induce apoptosis in A9-transfectants. Decreased levels of pro-survival marker Mcl-1, and increased levels of pro-apoptotic markers cleaved PARP and cleaved caspase-3 validated induction of apoptosis by SF + MU in EV-transfectants; no changes in these markers were observed in A9-transfectants (Fig. 4c, d).

### Inhibition of motility and invasion by SF + MU is attenuated in A9-transfectants

**Fig. 5:**
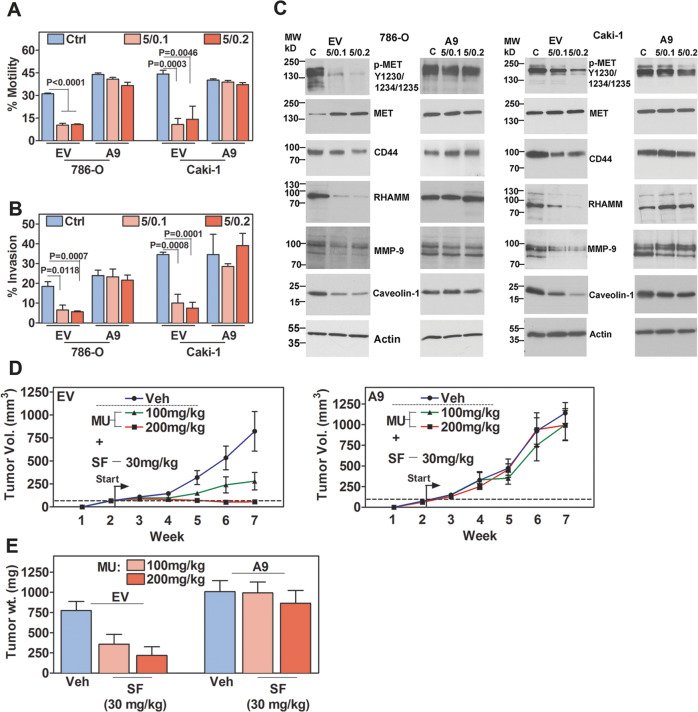
Effect of SF + MU treatment on invasive activities and tumor growth. **a**, **b** EV and A9 transfectants were treated with SF + MU (5/0.1, 5/0.2). Chemotactic motility and invasion were determined after 18- and 48-h, respectively. Data: mean ± SD (*n* = 3). **c** Immunoblot analysis following 48-htreatment. Loading control: actin. **d**, **e** Caki-1 subcutaneous xenograft: Mice implanted subcutaneously with Caki-1 EV or A9 transfectants were treated daily with vehicle (Veh) or SF + MU combination at indicated doses, starting on day 14 (start). **d** Tumor volume; **e** Tumor weight. Data in **d**, **e** mean ± SEM (*n* = 6/group; 2 males and 4 females each)).

In our previous study, we demonstrated that SF + MU effectively inhibited RCC cell motility and invasion^26^. Therefore, we evaluated the effect of SF + MU treatment on chemotactic motility and invasive activity of EV- and A9-transfectants. Consistent with previous results, SF + MU inhibited chemotactic motility by 3-fold and invasion by 4-fold in EV-transfectants, compared to untreated control (Fig. 5a, b). However, in A9-transfectants, SF + MU inhibited chemotactic motility only by 1.2-fold, with no inhibition of invasive activity (Fig. 5a, b). Additionally, when compared to Ctrl-shRNA transfectants, SF alone inhibited wound closure and invasive activity of A9-shRNA transfectants (Supplementary Fig. 3A–D).

We have previously shown that HA receptors CD44 and RHAMM are elevated in RCC specimens and that their expression correlates with metastasis^40^. CD44 and RHAMM have been shown to complex with MET, and promote an invasive phenotype including up-regulation of MMP-9 and Caveolin-1 expression^22,23,41,42^. Consistently, in EV-transfectants SF + MU downregulated CD44, RHAMM, phospho-MET, MMP-9, and Caveolin-1 levels by 2–10-fold (Fig. 5c, Supplementary Table 1). However, SF + MU did not significantly downregulate their levels in A9-transfectants of both cell lines (Fig. 5c).

### A9 expressing Caki-1 tumors are resistant to SF + MU: subcutaneous model

Caki-1 tumors are resistant to SF treatment at 60-mg/kg, which is close to the maximum tolerated dose^43^. Previously, we showed that SF + MU combination inhibited Caki-1 tumor growth in a subcutaneous xenograft, without serum or tissue toxicity^26^. Treatment of Caki-1 EV tumors with SF (30 mg/kg) and MU (100 or 200 mg/kg), starting when tumors reached ~ 100 mm^3^, inhibited tumor growth. When compared to the vehicle group, tumor weight decreased by ~ 80% in SF + MU treatment groups, however, A9 tumors were resistant to the treatment (Fig. 5d, e). SF + MU treatment did not affect animal weight (Supplementary Fig. 3E).

**Fig. 6:**
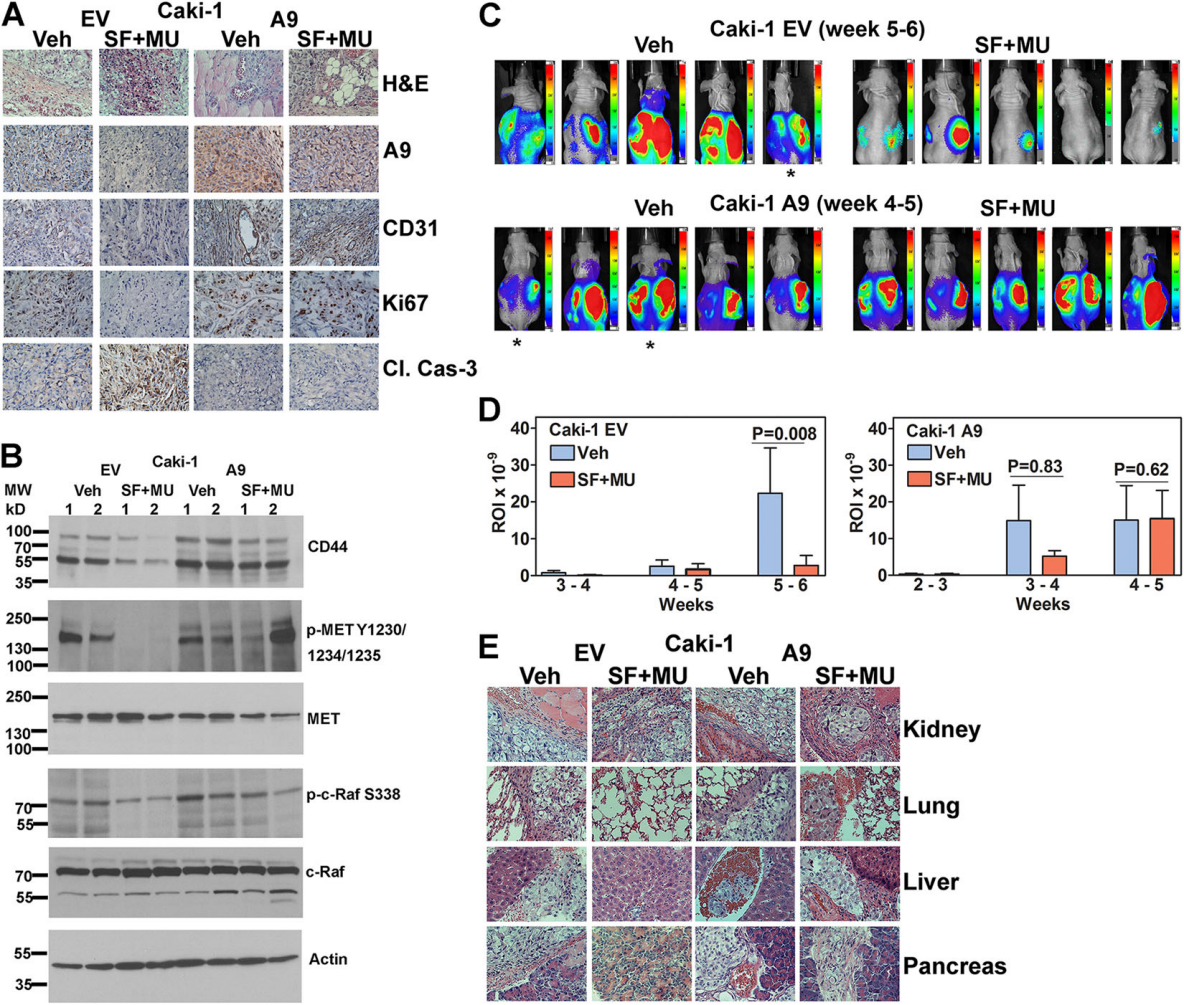
Effect of SF + MU treatment on tumor growth and metastasis. **a** Histology and IHC analyses of tumor specimens from Caki-1 subcutaneous model; magnification: ×400. **b** Immunoblot analysis of tumor tissues from vehicle or treatment groups. Tumor specimens were obtained from two animals per treatment group (labeled as 1 and 2). Loading control: Actin. **c**–**e** Orthotopic kidney (EV and A9) xenografts were generated and mice were treated with vehicle (Veh) or SF + MU (SF: 30 mg/kg; MU: 200 mg/kg). **c** Bioluminescence images for all groups at indicated time. **d** Quantification of luciferase signal using AMIView software. Data: mean ± SEM (*n* = 5; males). **e** Histology of kidney and other indicated organs.

EV and A9 tumors in both the vehicle and treatment groups were angiogenic and invaded skeletal muscle, microvessels, and subcutaneous fat. However, EV tumors in the treatment group displayed pyknotic nuclei (Fig. 6a). As expected A9 was downregulated in the SF + MU treated EV tumors but the treatment did not affect the expression in A9 tumors (Fig. 6a). SF + MU treated EV tumors were devoid of microvessels and Ki67 staining (proliferation index), but were positive for active (cleaved) caspase-3 staining. Vehicle-treated EV tumors and vehicle or SF + MU treated A9 tumors showed high microvessel density and Ki67 staining but low cleaved caspase-3 expression (Fig. 6a, Supplementary Fig. 3F, G). Further analysis of tumor tissues, confirmed the in vitro results that when compared to the vehicle-treated group, SF + MU treatment downregulated phospho-MET, and CD44 levels in EV tumors, but not in A9 tumors (Fig. 6b). SF is known to potently inhibit the kinase activity of c-Raf; IC_50_ of 6 nmol/L^44^. In SF + MU treated EV tumors, phospho-c-Raf (S338) levels were downregulated by 2.5-fold, whereas, the levels were not consistently affected in A9 tumors (Fig. 6b).

### A9 expression attenuates antitumor and antimetastatic efficacy of SF + MU: orthotopic model

For the orthotopic model, we used luciferase-expressing Caki-1 cells (Caki-1-luc). In the vehicle treatment group, Caki-1-luc EV tumors developed within 4–5 weeks post-surgery and metastasis was visible at 5–6 weeks (end point; Fig. 6c). In the SF + MU group, 80% of mice developed tumors by 5–6 weeks but tumor growth was significantly slower and 80% of the mice did not show visible metastasis in organ histology (Fig. 6c, e).

The bioluminescence intensity of the EV tumors in the treatment group was 32-fold lower than in the vehicle group at 5–6 weeks (*P* = 0.008; Fig. 6d). All mice implanted with A9-transfectant developed tumors within 3 weeks and distant metastasis by 4–5 weeks. A9 tumors were resistant to treatment (Fig. 6c). Histology confirmed primary kidney tumor and metastasis to lungs, liver and pancreas in the EV-vehicle and in A9-vehicle and treatment groups (Fig. 6e). In the EV-treatment group, histology confirmed a small kidney tumor but metastasis was abrogated (Fig. 6e).

These results demonstrate that the expression of A9 in RCC cells is responsible for SF resistance. However, downregulation of A9 by MU sensitizes cells to SF treatment. Furthermore, SF + MU combination has potent antitumor and antimetastatic efficacy without toxicity.

## Discussion

Despite several new classes of targeted agents being approved for therapy, treatment resistance is a major challenge that continues to drive the dismal five-year survival of mRCC patients^1–3^. In addition to discovering new therapeutic agents, understanding why a drug fails may lead to strategies to overcome drug resistance. While pathways/targets such as, ERK, EGFR, PI3K/Akt, hypoxia, autophagy, and EMT have been implicated in SF failure, none are targets of SF, nor do they target SF for metabolism/inactivation. These pathways also do not reveal why some patients respond to SF treatment while others do not^15,16,45^. Our study demonstrates, for the first time, that upregulation of A9 in tumor tissues, may at least be one of the mechanisms contributing to SF failure in the clinic. This is because glucuronidation of SF by A9 is the terminal inactivating SF biotransformation^32,33,35^. By downregulating A9, MU synergizes with SF to effectively abrogate RCC growth and metastasis. The salient points of our study are as follows: 1. A9 levels are elevated in RCC tissues/cells and potentially predict metastasis in RCC patients. 2. RCC cells are able to glucuronidate SF. 3. By downregulating A9, MU blocks inactivation of SF, and consequently, improves its antitumor and antimetastatic efficacy. 4. Since the combination is effective at low doses, where both drugs individually are ineffective, it should minimize off-target effects and toxicity.

Our results demonstrate that SF would have modest efficacy when used as a treatment for mRCC. This is because A9 levels are significantly elevated in tumors from patients who either have or will develop metastasis and SF is used for treating mRCC. Data on RCC cell lines show that A9 levels are highly upregulated in RCC cells when compared to NK epithelial cells. Upregulation of A9 in RCC cells was rather unexpected, since A9 is the major UGT enzyme in the kidney and a study on 26 kidney specimens reported downregulation of A9 in kidney tumors when compared to NK tissues^38^. However, in a clinical cohort of 134 specimens our study demonstrates that A9 levels are highly elevated in different types of RCC, as compared to NK and oncocytoma. Distinguishing between small renal tumors (<4 cm) and oncocytoma is clinically significant^46^. Since A9 levels in small renal tumors are ~16-fold higher than in oncocytoma, increased A9 levels may be of value in percutaneous biopsy tissue. At present, it is unclear why A9 is upregulated in invasive tumors. It is possible that detoxification of metabolic byproducts by terminal A9-mediated glucuronidation enhances tumor cell survival. Since mRCC is rarely biopsied, we could not analyze A9 expression in metastatic tissues. Nevertheless, increased A9 expression in RCC tumors that metastasize implies that mRCC may be inherently less sensitive to SF treatment. This is further corroborated by the ccRCC TCGA cohort in which A9 expression correlates with M-stage and is a predictor of OS.

Although MU is known to inhibit HA synthesis^20,22–25^, downregulation of A9 by MU at doses where MU does not inhibit HA synthesis reveals that A9 is the target of MU at low doses. A9 expression under a viral promoter attenuates the inhibitory effects of SF + MU against RCC cells in both *in vitro* and xenograft models. This further establishes that A9 downregulation by MU is a key reason for the observed anti-RCC efficacy of the SF + MU combination. Re-sensitization of RCC cells to SF alone by shRNA-mediated downregulation of A9 is again supportive of A9-overexpression plausibly contributing to SF unresponsiveness in RCC cells and mRCC.

SF + MU combination inhibited the growth and invasive activities of RCC cells and of endothelial cells co-cultured with RCC cells. Furthermore, ectopic expression of A9 not only attenuated the inhibitory effects of the combination in RCC cells, but also protected endothelial cells from these effects. This suggests that by overexpressing A9, RCC cells ensure an angiogenic microenvironment that is resistant to SF treatment. Furthermore, the increased efficacy of SF due to A9 downregulation is the basis for the high efficacy of SF + MU in preclinical models of RCC. Indeed, tumors in the SF + MU treatment groups grew only about 100–200 mm^3^, the size beyond which tumors require angiogenesis for growth and dissemination. SF + MU also inhibited the growth of patient-derived tumorspheres. This demonstrates that assessment of the efficacy of SF + MU in patient-derived tumorspheres together with the evaluation of A9 protein expression in tumor microsomes, could be exploited for clinical translation of the combination.

Effective treatments that directly target drug resistance could improve the outcome of mRCC patients. The orthotopic Caki-1-luc model has 100% tumor-take and with distant organ metastasis developing within 5–6 weeks. RCC primarily metastasizes via venous circulation, with frequent sites of metastases being lung, bone, lymph node, and liver; atypical sites include adrenal glands, brain, and pancreas^47^. In the Caki-1-luc model, tumors metastasized to lungs, liver, and pancreas. In this model bone metastasis was not visible, probably because the experimental end point (5–6 weeks) due to large kidney mass, was reached prior to frank bone metastasis. In this aggressive model, SF + MU oral treatment slowed tumor growth and abrogated metastasis in the majority of animals. This demonstrates that SF + MU may be effective as an antimetastatic treatment. The unresponsiveness of A9 tumors to the combination further demonstrates that A9 downregulation is a key reason for the high efficacy of SF + MU in RCC models.

Downregulation of A9 by MU raises the possibility that the combination may be associated with increased SF-related toxicity. However, SF is primarily metabolized by the CYP3A4 pathway in the liver^32,33,35^. Toxicity may also be less of a concern since due to synergy, lower doses of SF and MU are needed to achieve therapeutic response. Moreover, in both the present and our published studies, SF + MU did not cause serum or tissue toxicity and mice did not lose weight^26^. Since SF is FDA-approved and MU is available as OTC-supplement, their combination is potentially a targeted, minimally toxic, and effective treatment against mRCC. Broadly, our study highlights how targeting specific mechanisms that cause the failure of “old” modestly effective FDA-approved drugs, could improve treatment responsiveness in cancer patients with minimal alteration in toxicity profile.

## Materials and methods

### Cell lines and reagents

Human RCC cell lines (786-O, Caki-1, and 769-P), immortalized normal kidney cell line (HK-2) and human dermal (HMEC-1) and lung (HULEC-5a) microvessel endothelial cells were obtained from American Type Culture Collection® and cultured as per ATCC recommendations. Cell lines were authenticated and tested for mycoplasma contamination by Genetica DNA Laboratories Inc., Cincinnati, OH. All experiments were conducted within ten passages. Reagents, primers, constructs and antibodies are described in Supplementary Table 6.

### Clinical specimens and tumorspheres: clinical-cohort

Eighty-three RCC and 51 normal kidney (NK) specimens were obtained from patients undergoing nephrectomy for RCC (Supplementary Table 2). Specimens were obtained at University of Miami, Miller School of Medicine under an approved institutional review board protocol and after obtaining informed patient consent. De-identified specimens and de-linked data were transferred to Augusta University under an approved protocol. All clinical specimens are consecutively numbered such that investigators performing the assays were blinded from clinical information. Analysis was performed after all samples were tested. Tumorspheres were established from fresh clinical specimens collected at Augusta University under an approved protocol. Tumorspheres were established under ultra-low attachment conditions in MammoCult^TM^ Medium. For proliferation assays primary cultures were either plated in 2D adherent or 3D ultra-low attachment conditions and treated 24 h later.

### TCGA cohort

TCGA data on 542 clear cell RCC specimens was accessed through UCSC-Xena Browser and included demographic/pathologic parameters, overall survival (OS) and RNA-Seq data. Since the UGT1A 8–10 isoforms have 96% nucleotide sequence identity, A9 probes should recognize other isoforms. Therefore, data of all three isoforms was utilized for reporting the association of A9 clinical outcome.

### Glucuronidation assay

In all, 786-O cells cultured in growth medium were incubated with MU (0.2 mM) for 8.5 h followed by incubation with SF for 12 h. The cells and media were extracted in equal volume of acetonitrile, followed by extraction in ethylacetate. Sorafenib control was extracted 5 min after adding on the cells. SF was also incubated with UGT1A9 supersomes at 37 °C for 25 min in an UGT assay buffer (BD Biosciences). Ethylacetate extracts of all samples were dried, resuspended in acetonitrile, and subjected to reverse phase HPLC on a C18 column; gradient: acetonitrile and 20 mM ammonium acetate/0.1% formic acid^29,33^.

### Microsome preparation

Microsomes were prepared as described by Mohr et al.^48^. Microsomes were characterized by immunoblot analysis of microsome marker, cytochrome p450 oxidoreductase (POR).

### A9-overexpression and knockdown

Full length human A9 cDNA (Genbank: NM_021027) was cloned into the pQCXIH retroviral expression vector with a 3×-FLAG tag at the C-terminus; EV: empty vector with no insert. 786-O and Caki-1 cells were stably transfected with EV or A9 construct by retroviral infection. For A9 knockdown, RCC cells were transfected with A9-shRNA (Supplementary Table 6) or a non-targeting shRNA.

### Phenotypic readout assays for EV and A9 transfectants

Proliferation: transfectants (5×10^4^ cells/well) cultured in growth medium were treated with SF (0–15 µM) and MU (0–0.2 mM) combination; viable cells were counted at 72-hours.

Colony assay: transfectants (500 cells/well) were treated with SF + MU for 10 days. Colonies containing ≥50 cells were stained with crystal violet and counted.

Co-culture studies: in a 2D-assay, transfectants (bottom chamber) were co-cultured with HMEC-1 or HULEC-5a (top chamber; 3 µm insert). 24-h later co-cultures were treated with SF + MU for 48-hours. Cell viability was assessed by MTT (3-(4, 5-dimethylthiazolyl-2)-2,5 diphenyltetra-zolium bromide) assay.

Motility and invasion: transfectants were treated with SF + MU, and motility and invasion were assessed after 18- and 48-h incubation, respectively^26^. In a scratch wound assay, 786-O cell transfectants were cultured in 0.1% FBS containing medium. Wound closure was calculated as described before^23^.

Cell cycle analysis and apoptosis: transfectants were treated with SF + MU for 24- (cell-cycle) or 48-h (apoptosis). Cell cycle was analyzed by flow cytometry following propidium iodide staining and using ModFit LT v4 software. Apoptosis was measured using a Cell Death Detection ELISA^PLUS^ Kit.

### RT-qPCR and immunoblot assays

786-O and Caki-1 transfectants treated with SF + MU for 48–60 h. Total RNA or cell lysates were subjected to reverse-transcription quantitative Polymerase Chain Reaction (RT-qPCR) or immunoblotting, respectively, (Supplementary Table 6).

### Xenograft studies

All studies on mice were conducted using a protocol approved by the Institutional Animal Care and Use Committee. Animals were randomized into vehicle or treatment groups based on the order of retrieval from cages. Cages were housed in random order on shelves. Investigators preparing drug combinations did not administer the drugs.

### Subcutaneous xenograft

Dorsal flanks of 5–6 week old athymic nude mice were subcutaneously injected with 2 × 10^6^ Caki-1 cells mixed 1:1 with Matrigel^TM^. Treatment was started when tumors reached ~100 mm^3^ (day 14, ref. ^2^). SF was dissolved in Koliphor EL and ethanol solution (1:1); MU was dissolved in filter-sterilized 2% sucrose. SF and MU solutions (1:4 proportion; final ethanol concentration 12.5%) were mixed and mice were gavaged daily with 0.1 ml volume of SF + MU Tumor volume was measured weekly (end point: 49 days; tumor volume ~1000 mm^3^).

### Orthotopic model

Luciferase-expressing Caki-1 transfectants (EV, A9) were implanted underneath the renal capsule of 8-week-old athymic mice. From day 9, mice were treated with SF + MU or the vehicle and imaged weekly using Ami-X imaging system (Spectral Instruments Imaging). Images were analyzed using AMIView Software.

### Histology and immunohistochemistry

Tumors and organs were analyzed by histology. Tumor specimens were stained for microvessels (anti-CD31), A9 and Ki67 (proliferation index) as described before^49^.

### Sample size calculation

A9 levels were measured on 134 available clinical specimens. The mean difference in A9 levels between normal (0.97 ± 1.8) and tumor (4.9 ± 7.1) specimen was 3.93. To detect this difference with 80% power we only needed a total of 54 specimens With 134 specimens, our study was sufficiently powered. In the xenograft studies, the mean difference in tumor weights between the vehicle (774.5 ± 274.6) and the treatment group (218.5 ± 261.5) was 556. To detect this difference with 80% power we only needed a total of 8 animals (or 4 animals per group). With a total of 10 or 12 animals in xenograft models, our study was sufficiently powered.

### Statistical analyses

JMP Pro 14 and GraphPad Prism 8.0.0 software were used for analyses. No samples were excluded from the analysis. In the clinical-cohort, the significance of differences in the expression of A9 between groups were evaluated by one-way ANOVA followed by Mann-Whitney U test because data were determined to be non-normally distributed as per the Shapiro Wilks’ test; *P*-values are two-tailed. Association of A9 expression with clinical and outcome parameters was determined by logistic regression and Cox proportional hazard models. Kaplan–Meier plots with log-rank statistics were prepared to determine if A9 expression categorized RCC patients into risk categories for predicting metastasis. For TCGA data, combined A9 marker was generated as described before^50,51^. Experiments were repeated in two different cell line models or primary tumor spheroids or two xenograft models as indicated. Mean ± SD (or SEM) was computed for quantifiable parameters (e.g., cell number, percentage motility, percentage invasion, and tumor volume). Differences among the transfectants were compared by one-way ANOVA followed by unpaired *t*-test (e.g., control versus treatment); *P*-values were two-tailed.

## Supplementary information

Supplementary Marerials
