# Supplementary material for: Molecular targeting of renal cell carcinoma by an oral combination
Source: Oncogenesis. 2020 May 19;9(5):52. doi: 10.1038/s41389-020-0233-0 (PMC7237463; doi:10.1038/s41389-020-0233-0)
Supplement: Supplementary file 1 — Supplementary Marerials [file 41389_2020_233_MOESM1_ESM.docx]

**Molecular Targeting of Renal Cell Carcinoma by an Oral Combination**

**Jordan and Wang et al**

**Supplementary Materials**

**Supplementary Figure 1: CYP3A4 expression and SF glucuronidation in cell lines. A**: Immunoblot analysis of HK-2 and RCC cell line lysates for CYP3A4 protein expression. **B**: Immunoblot analysis of CYP3A4 expression in 786-O and Caki-1 cells untreated (C: control) or treated with MU (0.2 mM), SF (5 μM) or SF+MU (5 µM SF and 0.2 mM MU). A and B: Loading control: actin **C**: Reverse-phase HPLC profile of SF and metabolite levels in 786-O EV and A9 transfectants treated with SF, or SF+MU. Ratio of the area under the curve of the first and the second peaks showed that SF+MU treatment caused about 86% inhibition of SF glucuronidation in EV cells and about 40% inhibition in A9 cells. SF and SF assayed using A9 supersomes serve as controls (32).


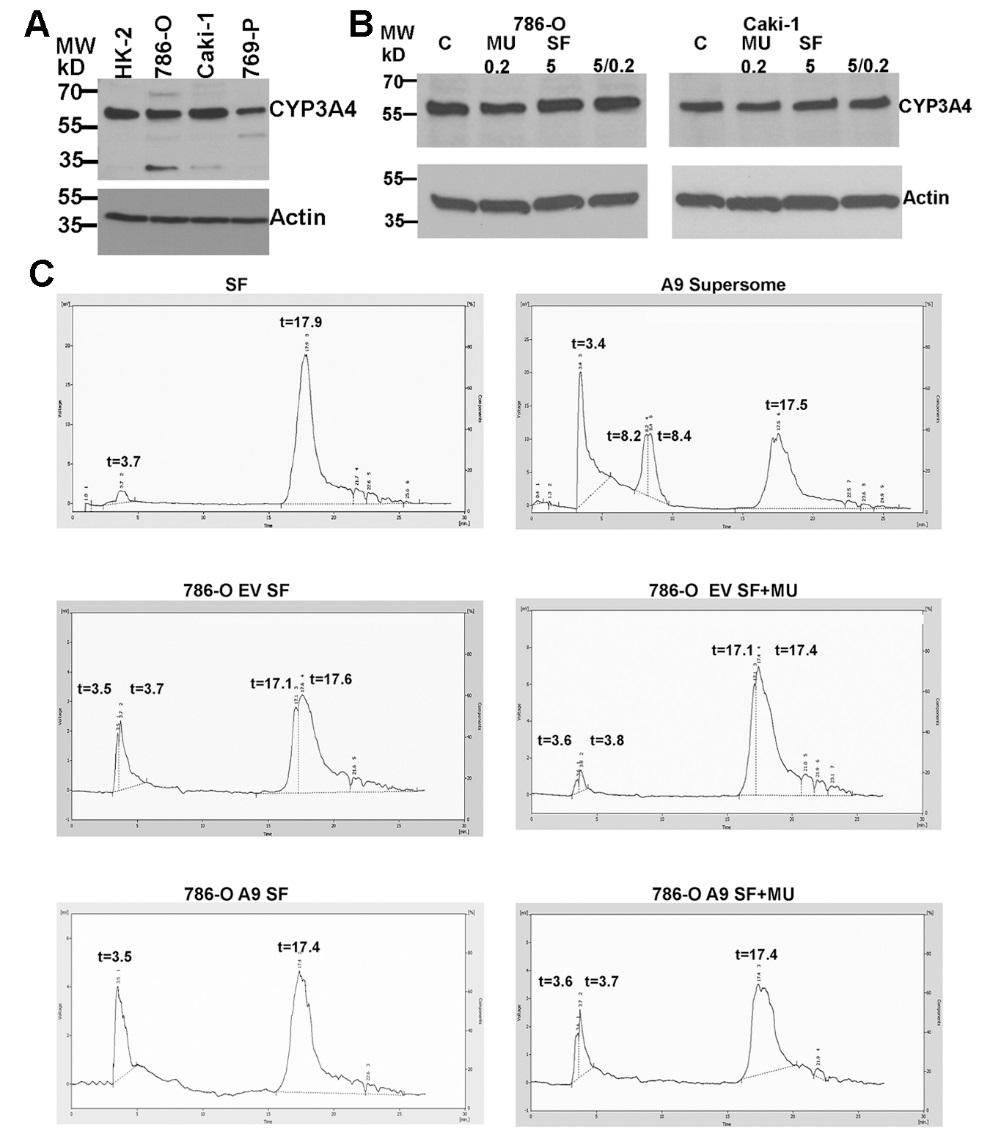


**Supplementary Table 1:** Immunoblot Relative Intensities: Relative intensities of the immunoblot data presented in various figures. For each sample the normalized value (Intensity of the protein of interest ÷ intensity of loading control) was obtained. The normalized values in a treatment sample (e.g., SF+MU doses: 5/.1, 5/.2) was divided by the corresponding normalized values in the control sample (or Veh) to obtain fold change values. Therefore, for the control, the fold change was equal to 1.

| **Figure 1A** | Samples | Fold changes |
| --- | --- | --- |
| A9 | HK-2 | 1 |
|  | 786-O | 18.95 |
|  | Caki-1 | 19.90 |
|  | 769-P | 14.91 |
|  | | |

| **Figure 1C** | Samples | | Fold changes |
| --- | --- | --- | --- |
| A9 | 786-O | C | 1 |
|  |  | MU 0.2 | 0.26 |
|  |  | SF 5 | 0.84 |
|  | Caki-1 | C | 1 |
|  |  | MU 0.2 | 0.34 |
|  |  | SF 5 | 1.01 |
|  | | | |

| **Figure 1E** | | | | | | |
| --- | --- | --- | --- | --- | --- | --- |
|  |  | **786-O EV** | |  | **786-O A9** | |
| **Protein** | C | 5/0.1 | 5/0.2 | C | 5/0.1 | 5/0.2 |
| A9 | 1 | 0.44 | 0.23 | 1 | 0.84 | 0.88 |
|  |  | **Caki-1 EV** | |  | **Caki-1 A9** | |
| **Protein** | C | 5/0.1 | 5/0.2 | C | 5/0.1 | 5/0.2 |
| A9 | 1 | 0.20 | 0.17 | 1 | 0.67 | 0.75 |

| **Figure 2E** | Samples | | Fold changes |
| --- | --- | --- | --- |
| A9 | NK | 1 | 1.00 |
|  |  | 2 | 0.73 |
|  |  | 3 | 0.70 |
|  | Non-Met | 1 | 0.81 |
|  |  | 2 | 5.66 |
|  |  | 3 | 4.50 |
|  | Met | 1 | 9.58 |
|  |  | 2 | 10.26 |
|  |  | 3 | 11.64 |

| **Figure 4C&D** | | | | | | |
| --- | --- | --- | --- | --- | --- | --- |
|  |  | **786-O EV** | |  | **786-O A9** | |
| **Protein** | C | 5/0.1 | 5/0.2 | C | 5/0.1 | 5/0.2 |
| Cyclin B1 | 1 | 2.48 | 6.50 | 1 | 1.12 | 2.74 |
| Cyclin E1 | 1 | 0.29 | 0.22 | 1 | 0.60 | 0.44 |
| p-CDK1 | 1 | 0.95 | 2.35 | 1 | 1.12 | 1.00 |
| CDK1 | 1 | 1.48 | 1.38 | 1 | 1.13 | 1.09 |
| p-Rb | 1 | 0.84 | 0.51 | 1 | 1.23 | 1.03 |
| Rb | 1 | 1.02 | 0.87 | 1 | 1.06 | 0.76 |
| Mcl-1 | 1 | 0.25 | 0.17 | 1 | 0.87 | 0.91 |
| Cl. Cas-3 | 1 | 1.40 | 3.54 | 1 | 0.79 | 1.07 |
| Cl. PARP | 1 | 1.64 | 1.67 | 1 | 1.34 | 1.01 |
|  |  | **Caki-1 EV** | |  | **Caki-1 A9** | |
| **Protein** | C | 5/0.1 | 5/0.2 | C | 5/0.1 | 5/0.2 |
| Cyclin D1 | 1 | 0.77 | 0.21 | 1 | 1.45 | 1.26 |
| Cyclin E1 | 1 | 0.36 | 0.37 | 1 | 1.06 | 1.57 |
| p-CDK2 | 1 | 1.09 | 0.76 | 1 | 1.19 | 1.05 |
| CDK2 | 1 | 1.22 | 1.03 | 1 | 1.56 | 1.75 |
| p21 | 1 | 4.85 | 5.29 | 1 | 1.42 | 0.92 |
| p-Rb | 1 | 0.82 | 0.04 | 1 | 1.41 | 1.10 |
| Rb | 1 | 1.03 | 1.11 | 1 | 1.36 | 1.12 |
| Mcl-1 | 1 | 0.95 | 0.12 | 1 | 1.08 | 1.00 |
| Cl. Cas-3 | 1 | 12.21 | 22.86 | 1 | 1.48 | 1.22 |
| Cl. PARP | 1 | 5.63 | 5.58 | 1 | 1.31 | 1.01 |
|  | | | | | | |
| **Figure 5C** | | | | | | |
|  |  | **786-O EV** | |  | **786-O A9** | |
| **Protein** | C | 5/0.1 | 5/0.2 | C | 5/0.1 | 5/0.2 |
| p-MET | 1 | 0.08 | 0.02 | 1 | 0.94 | 1.01 |
| MET | 1 | 1.61 | 1.28 | 1 | 1.02 | 0.91 |
| CD44 | 1 | 0.78 | 0.54 | 1 | 1.00 | 0.95 |
| RHAMM | 1 | 0.05 | 0.02 | 1 | 1.14 | 1.51 |
| MMP-9 | 1 | 0.28 | 0.32 | 1 | 0.87 | 0.82 |
| Caveolin-1 | 1 | 0.38 | 0.27 | 1 | 1.26 | 0.92 |
|  |  | **Caki-1 EV** | |  | **Caki-1 A9** | |
| **Protein** | C | 5/0.1 | 5/0.2 | 0 | 5/0.1 | 5/0.2 |
| pMET | 1 | 0.68 | 0.33 | 1 | 0.88 | 0.81 |
| MET | 1 | 1.10 | 1.16 | 1 | 0.96 | 0.98 |
| CD44 | 1 | 0.35 | 0.48 | 1 | 1.06 | 0.99 |
| RHAMM | 1 | 0.25 | 0.05 | 1 | 1.58 | 1.39 |
| MMP-9 | 1 | 0.30 | 0.22 | 1 | 0.86 | 0.87 |
| Caveolin-1 | 1 | 0.52 | 0.19 | 1 | 0.84 | 0.90 |

| **Figure 6E** | | | | | | | | |
| --- | --- | --- | --- | --- | --- | --- | --- | --- |
|  | **EV** | | | | **A9** | | | |
| Protein | **Veh**  **#1** | **Veh**  **#2** | **SF+MU #1** | **SF+MU #2** | **Veh #1** | **Veh #2** | **SF+MU #1** | **SF+MU #2** |
| CD44 | 1 | 0.80 | 0.27 | 0.21 | 1 | 1.20 | 1.05 | 1.05 |
| p-MET | 1 | 0.55 | 0.01 | 0.01 | 1 | 0.73 | 0.35 | 5.78 |
| MET | 1 | 0.83 | 0.74 | 0.52 | 1 | 1.28 | 0.87 | 0.88 |
| p-c-RAF | 1 | 1.00 | 0.43 | 0.38 | 1 | 0.75 | 0.61 | 0.44 |
| RAF | 1 | 0.72 | 0.63 | 0.63 | 1 | 1.14 | 1.05 | 1.21 |
|  |  |  |  |  |  |  |  |  |

| **Supplementary**  **Figure 1A** | Samples | Fold changes |
| --- | --- | --- |
| CYP3A4 | HK-2 | 1.02 |
|  | 786-O | 0.97 |
|  | Caki-1 | 1.30 |
|  | 769-P | 0.66 |
|  | | |

| **Supplementary Figure 1B** | Samples | | Fold changes |
| --- | --- | --- | --- |
| CYP3A4 | 786-O | C | 1 |
|  |  | MU 0.2 | 1.03 |
|  |  | SF 5 | 0.94 |
|  |  | 5/0.2 | 0.91 |
|  | Caki-1 | C | 1 |
|  |  | MU 0.2 | 1.01 |
|  |  | SF 5 | 0.85 |
|  |  | 5/0.2 | 0.85 |

| **Supplementary Figure 2A** | Samples | | Fold changes |
| --- | --- | --- | --- |
| A9 | 786-O | Ctrl shRNA | 1 |
|  |  | A9 shRNA #1 | 0.15 |
|  |  | A9 shRNA #2 | 0.17 |
|  | Caki-1 | Ctrl shRNA | 1 |
|  |  | A9 shRNA #1 | 0.20 |
|  |  | A9 shRNA #2 | 0.15 |

**Supplementary Table 2: Specimen and patient characteristics of clinical specimen cohort and the TCGA dataset.** The cohort consists of 134 specimens acquired from 83 RCC patients (51 normal kidney; 83 tumor specimens). TCGA ccRCC dataset consists of 542 tumor specimens. For age, Karnofsky score, and follow-up (metastasis; overall survival), Mean ± SD and median are reported. OS: (-) designates survival, (+) designates death.

| Parameter | Clinical cohort | TCGA cohort |
| --- | --- | --- |
| Number of specimens | Normal kidney: 51  Tumor: 83  RCC: 77; Oncocytoma: 6 | Tumor (RCC) = 542 |
| Age | 63.1 ± 14.1 yrs.; median: 64 yrs. | 60.6 ± 12.1 years; median 61 yrs |
| Gender | Male: 59; Female: 23; Unknown: 1 | Male: 351; Female: 191 |
| Tumor type | Clear cell: 58; Non-clear cell: Papillary: 10; Chromophobe: 5 Sarcomatoid: 2; Collecting Duct: 2; Oncocytoma: 6 | Clear cell: 542 |
| Tumor size | < 4 cm: 19; ≥ 4 cm: 62  Unknown: 2 | NA |
| Grade | Grade 0 (Oncocytoma): 6  Grade 1: 5; Grade 2: 29  Grade 3: 26 Grade 4: 16; Unknown: 1 | Grade 1:14; Grade 2: 234  Grade 3: 208 Grade 4: 78  Unknown: 8 |
| Stage | pT0: 6; pT1a: 19  pT1b: 18; pT2: 12  pT3a: 11 ; pT3b: 15 ; pT4: 1; Unknown: 1 | pT1: 165; pT1b: 113  pT2: 66; pT2b: 4  pT3: 127 pT3b: 56  pT4: 11 |
| Lymph node invasion | (-): 51; (+): 5;  Unknown: 27 | (-): 241; (+): 17;  Unknown: 284 |
| Lymphovascular invasion | (-): 43; (+): 7;  Unknown: 33 |  |
| Karnofsky score | (+) 36; Unknown: 47  Mean: 86.4 ± 14.2; median: 90 | (+): 28; Unknown: 514  Mean: 80.7 ± 34.6; 95 |
| Metastasis (clinical)  M-stage (TCGA) | (-): 64; (+): 18; Unknown: 1 | (-): 431; (+): 79  Unknown: 32 |
| Follow-up (Metastasis): | 33.1 ± 25.2 ; 27 months |  |
| Overall survival |  | (-): 363; (+): 177  Unknown: 2 |
| Follow-up (overall survival): |  | 44.1 ± 32.2; 38.6 months |

**Supplementary Table 3: Univariate analysis to determine relationship of clinical parameters and A9 levels to metastasis and OS**. Single parameter logistic regression was performed to evaluate the ability of clinical parameters and A9 expression to associate with metastasis in the clinical specimens or OS in the TCGA dataset. Units odds ratio (OR) and 95% CI (CI) are shown for significant parameters. *Logistic regression could not be computed for lymph node relationship to metastasis in the clinical specimen cohort due to all lymph node (+) patients progressing to metastasis.

|  | Clinical specimen cohort | | | TCGA data | | |
| --- | --- | --- | --- | --- | --- | --- |
|  | Metastasis | | | OS | | |
|  | χ^2^ | P value | OR; 95% CI | χ^2^ | P value | OR; 95% CI |
| Age | 1.01 | 0.315 |  | 22.13 | < 0.0001 | 1.04; 1.02 – 1.05 |
| Sex | 1.43 | 0.233 |  | 0.34 | 0.558 |  |
| Grade | 14.17 | 0.0002 | 5.06; 2.2 – 11.8 | 68.74 | < 0.0001 | 3.2; 2.4 – 4.2 |
| T-stage | 9.13 | 0.003 | 2.36; 1.35 – 4.12 | 65.56 | < 0.0001 | 2.33; 1.9 – 2.87 |
| Lymph node (+/-)* |  | | | 7.29 | 0.0069 | 4.4; 1.5 – 12.92 |
| Lymphovascular invasion (+/-) | 1.5 | 0.221 |  |  | | |
| M-stage |  | | | 67.3 | < 0.0001 | 12.5; 6.82 – 22.7 |
| Tumor size | 7.20 | 0.007 | 1.23; 1.06 – 1.44 |  | | |
| Renal Vein | 1.08 | 0.299 |  |  | | |
| A9 | 5.12 | 0.0236 | 1.07; 1.01 – 1.14 | 30.61 | <0.0001 | 2.82; 1.96 – 4.08 |

**Supplementary Table 4: Multivariate analysis to determine relationship of clinical parameters and A9 levels to metastasis and OS**. Cox Proportional Hazards Model was used to evaluate the ability of clinical parameters and A9 expression to associate with metastasis in the clinical cohort and with OS in TCGA cohort. Parameters included: Age, sex, tumor size, grade, stage, lymphovascular invasion, renal vein invasion, A9. Renal vein invasion and tumor size data were not available in TCGA dataset but M-stage was included. Only the parameters that reached significance are shown.

|  | Clinical specimen cohort | | | TCGA data | | |
| --- | --- | --- | --- | --- | --- | --- |
|  | Metastasis | | | OS | | |
| Parameter | χ^2^ | P-value | Range Hazard Ratio, 95% CI | χ^2^ | P-value | Range Hazard Ratio, 95% CI |
| age |  |  |  | 8.01 | 0.0046 | 1.02; 1.00 – 1.04 |
| T-stage | 8.06 | 0.005 | 23.71; 2.84 – 246 |  |  |  |
| M-stage |  |  |  | 15.02 | 0.0001 | 2.94; 1.7 – 5.08 |
| UGT1A9 | 4.40 | 0.036 | 1.08; 1.0 – 1.16 | 4.78 | 0.0287 | 1.71; 1.05 – 2.76 |

**Supplementary Table 5: IC_50_ values for growth inhibition in transfectants by SF alone or in combination with MU.** IC_50_ values were calculated based on the non-liner regression analysis of dose response curves using sigmoidal dose-response (variable slope) equation.

| **Transfectant** | **SF IC_50_ for transfectants** | | |
| --- | --- | --- | --- |
|  | **0 mM MU** | **0.1 mM MU** | **0.2 mM MU** |
| 786-O EV | 7.852 | 4.205 | 2.021 |
| 786-O A9 | 9.301 | 8.635 | 7.514 |
| Caki-1 EV | 7.757 | 4.863 | 2.179 |
| Caki-1 A9 | 9.581 | 8.265 | 6.854 |
|  | | | |
|  | **SF IC_50_ for shRNA transfectants** | | |
|  | Ctrl | A9 # 1 | A9 # 2 |
| 786-O | 7.899 | 3.492 | 3.215 |
| Caki-1 | 7.801 | 2.495 | 2.619 |

**Supplementary Figure 2: Analysis of Flag-tag A9 expression and shRNA transfectants. A.**  Immunoblot analysis of A9 expression in control (Ctrl) and A9 shRNA transfectants. Actin: loading control. **B** - **D:** Ctrl- and A9-shRNA transfectants of 786-O (B) and Caki-1 (C) were treated with SF, MU, or SF+MU at indicated doses. Viable cells were counted at 72-hours. Colonies were counted on day 7 (D). **E:** Viability of endothelial cells (HMEC-1; HULEC-5a) in co-cultures with Caki-1 transfectants following SF+MU treatment as measured by MTT-assay. Data in **B - E**: Mean ± SD (n=3 to n=8).


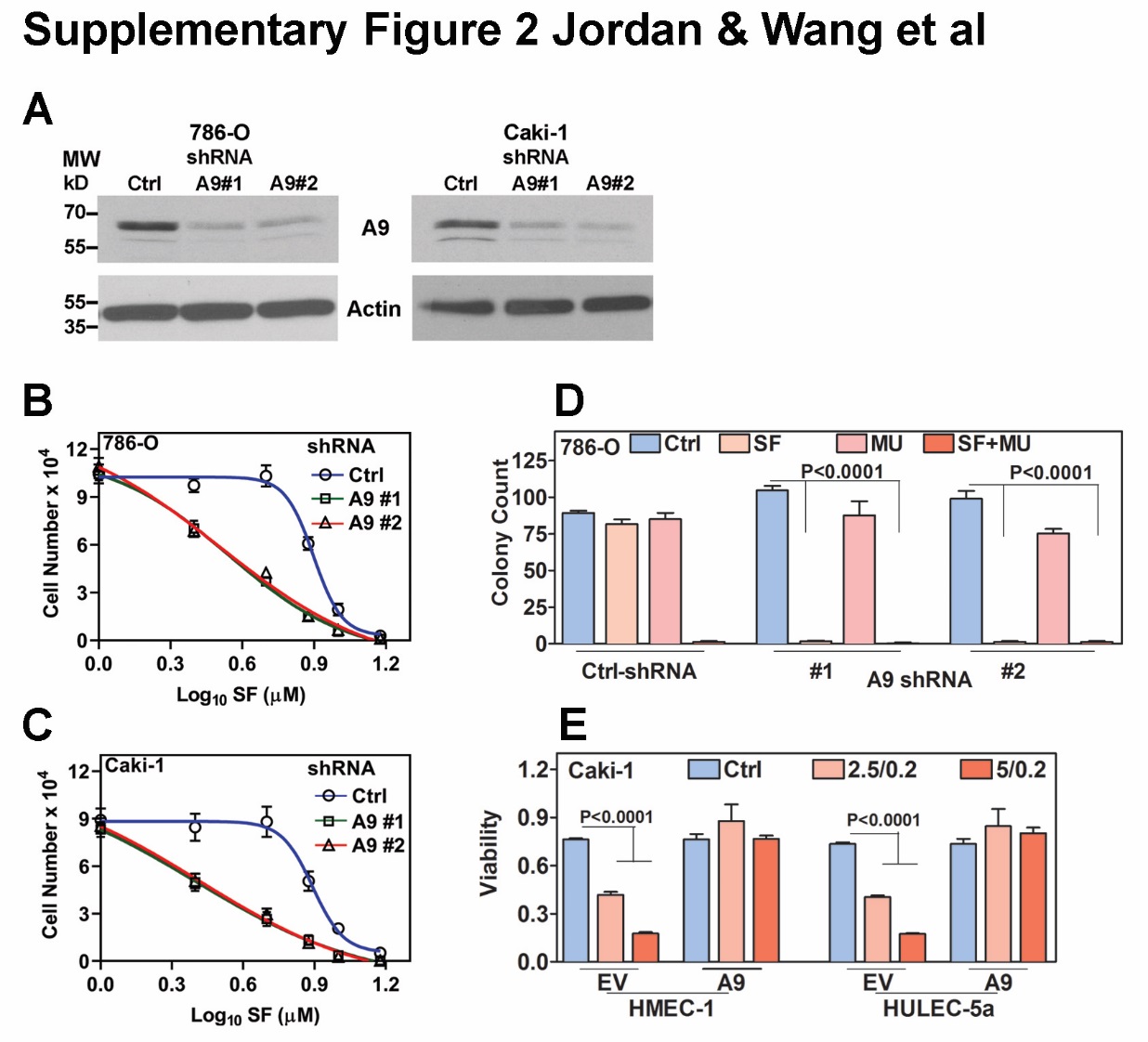


**Supplementary Figure 3: Effect of SF+MU on motility, invasion and Caki-1 xenograft. A**-**D:** 786-O Ctrl- and A9-shRNA transfectants were untreated (Ctrl) or treated with SF (5 μM), MU (0.2 mM), or SF+MU (5/0.2). At indicated times, % wound closure (A - C) and at 48-hours % invasion (D) were determined. Data: Mean ± SD (n = 3). **E:** Animal weight for Caki-1 subcutaneous model data presented in Figure 5. Data: Mean ± SD. **F and G:** Quantification of MVD and Ki67 positive nuclei (proliferation index) per high power field. Data: Mean ± SD; n = 10.


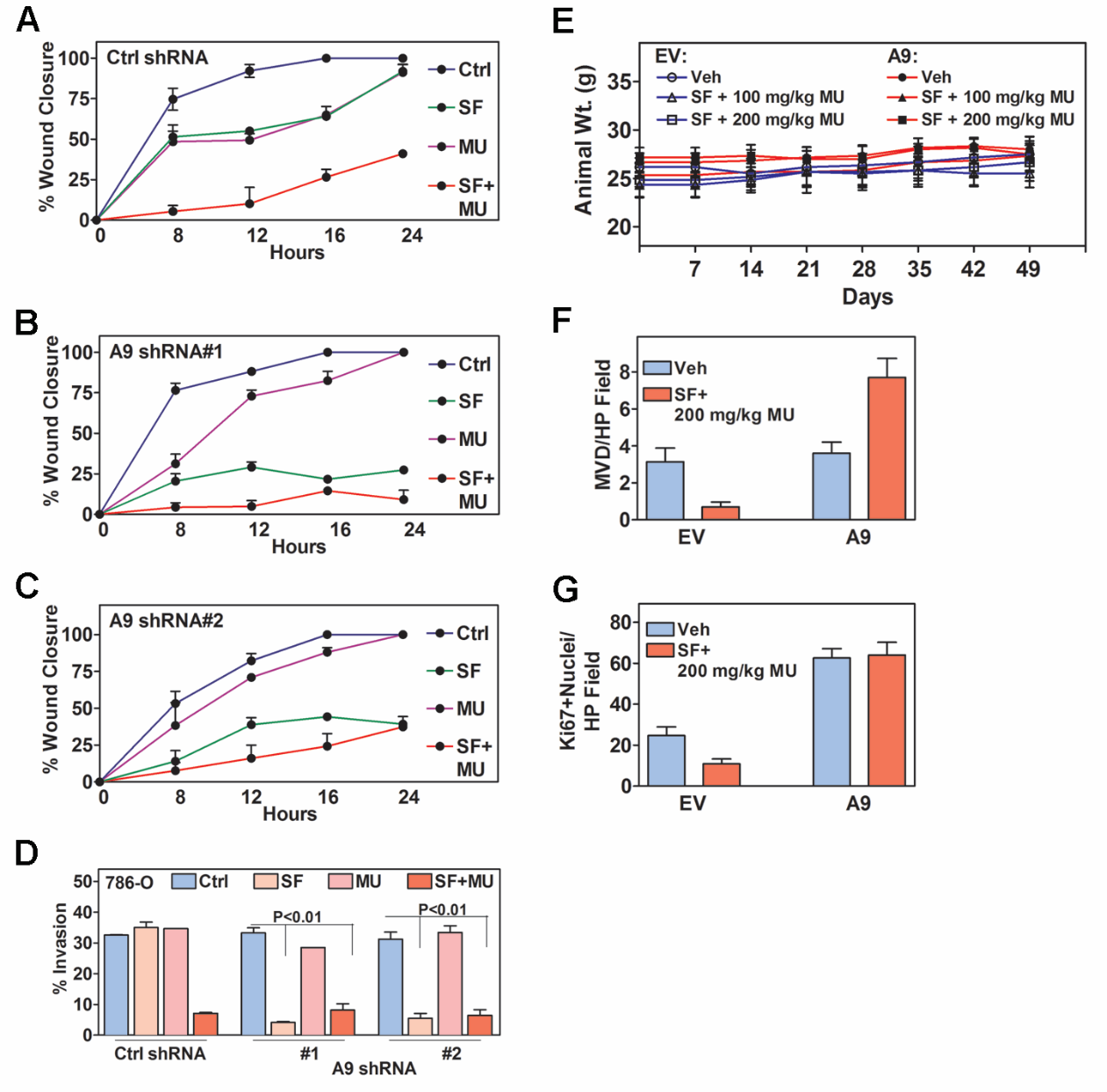


**Supplementary Table 6:** **Materials used in the study.** Description of reagents, antibodies and primers used in the study.

| **Antibodies used in Western Blot analysis** | | | | | | |
| --- | --- | --- | --- | --- | --- | --- |
| **Target** | **Host species** | **Supplier** | **Catalog** | **Clone** | **Lot #** | **Dilution** |
| Actin | Goat | Santa Cruz Biotechnology | sc-1615 HRP | C11 | J0914 | 1:20,000 |
| Cleaved Caspase-3 | Rabbit | Cell Signaling Technology | #9661 | D175 | 43 | 1:1,000 |
| Caveolin-1 | Rabbit | Cell Signaling Technology | #3267S | D64G3 | 2 | 1:2,500 |
| HCAM | Mouse | Santa Cruz Biotechnology | SC-7297 HRP | DF1485 | F0816 | 1:500 |
| CD44v6 | Mouse | R&D Systems | BBA13 | 2F10 | 5781 | 1:1,000 |
| Cdk1 Ab-3 (Cocktail) | Mouse | Neomarkers | MS-275-P1 | A17.1.1+POH-1 | 275P212C | 1:1,000 |
| Phospho-cdc2 (Tyr15) | Rabbit | Cell Signaling Technology | #4539 | 10A11 | 2 | 1:1,000 |
| Cdk2 Ab-4 | Mouse | Neomarkers | #MS-617-P1ABX | 2B6 + 8D4) | 617X310A | 1:2,000 |
| Phospho-CDK2 (Thr160) | Rabbit | Cell Signaling Technology | #2561 | Polyclonal | 2 | 1:1,000 |
| Cyclin B1 | Mouse | Santa Cruz Biotechnology | sc-245 | GNS1 | G0819 | 1:7,500 |
| Cyclin D1 | Rabbit | Abcam/Epitomics | 1677-1(ab40754) | EP272Y | GR106612-1 | 1:2,000 |
| Cyclin E1 | Rabbit | Epitomics | 3327-1(ab133266) | EPR194 | YH052101C | 1:2,000 |
| FLAG | Mouse | Sigma-Aldrich | F1804 | M2 | SLBF6631 | 1:2,000 |
| Mcl-1 | Rabbit | Epitomics | #1239-1 | D2W9E | 1 | 1:1,000 |
| Met (NT) | Rabbit | EMD Millipore | 04-1051 | EP1454Y | NG1944105 | 1:5,000 |
| Phospho-Met (Tyr1230/Tyr1234/Tyr1235) | Rabbit | MilliporeSigma | 07-810 | Polyclonal | 2766394 | 1:5,000 |
| MMP-9 | Rabbit | Epitomics | #2551-1 | EP1254 | YF-08-29-09C | 1:3,000 |
| p21 Waf1/Cip1 | Mouse | Cell Signaling Technology | #2946 | DCS60 | 5 | 1:2,000 |
| Cleaved PARP (Asp214) | Rabbit | Cell Signaling Technology | #9541 | D64E10 | 8 | 1:1,000 |
| POR | Rabbit | Abnova | H00005447 | B01P | 10146 | 1:1,500 |
| RAF1 (c-Raf) | Rabbit | ABclonal | A0223 | Polyclonal | 0014820201 | 1:2,000 |
| Phospho-RAF1-S338 | Rabbit | ABclonal | AP0498 | Polyclonal | 2101590101 | 1:1,000 |
| Rb (4H1) | Mouse | Cell Signaling Technology | 9309 | 4H1 | 9 | 1:2,000 |
| Phospho-Rb (Ser807/811) | Rabbit | Cell Signaling Technology | 9308 | Polyclonal | 12 | 1:1,000 |
| RHAMM (CD168) | Mouse | Novocastra | NCL-CD168 | 2D6 | 6021453 | 1:300 |
| UGT1A | Rabbit | GeneTex | GTX114128 | C1C3 | 40156 | 1:1,000 |
| CYP3A4 | Rabbit | ABclonal | A13843 | Polyclonal | 0006270101 | 1:1,000 |
|  |  |  |  |  |  |  |
|  | | | | | | |

| **Antibodies used in IHC** | | | | | | | |
| --- | --- | --- | --- | --- | --- | --- | --- |
| **Target** | **Host species** | **Supplier** | **Catalog** | | **Clone** | **Lot#** | **Dilution** |
| Ki67 | Rabbit | Abcam | Ab16667 | | SP6 | GR3228859-3 | 1:240 |
| CD31 | Rabbit | Abcam | Ab28364 | | Polyclonal | 6R272058-5 | 1:120 |
| UGT1A antibody | Rabbit | GeneTex | GTX114128 | | Polyclonal | 1 | 1:1,000 |
|  | | | | | | | |
| **Other Reagents** | | | | | | | |
| **Reagents and Kits** | | | | **Supplier** | | **Catalog #** | |
| RNeasy Mini Kit | | | | QIAGEN | | 74104 | |
| iScript^TM^ cDNA Synthesis Kit | | | | BIO RAD | | 1708891 | |
| SsoFast^TM^ Evagreen® Supermix | | | | BIO RAD | | 1725204 | |
| RPMI 1640 Medium | | | | Fischer Scientific | | MT10040CV | |
| MCDB131 Medium | | | | ThermoFischer Scientific | | 10372-019 | |
| Mammocult^TM^ Human Medium Kit | | | | STEMCELL Technologies | | 05620 | |
| Sorafenib, p-toluenesulfonate salt | | | | LC Laboratories | | SC-8502 | |
| 4-Methylumbelliferone sodium salt | | | | Sigma-Aldrich | | M1508 | |
| Cell Death ELISA^PLUS^ Kit | | | | **CELLDETH-RO**Roche; Sigma-Aldrich | | 11774425001 | |
| Crystal Violet | | | | Sigma-Aldrich | | C0775 | |
| Propidium Iodide | | | | Sigma-Aldrich | | P1470 | |
| Thiazolyl Blue Tetrazolium Bromide | | | | Sigma-Aldrich | | M5655 | |
| Cell Death Detection Elisa | | | | Roche Diagnostics GmbH | | 11544675001 | |
| Corning^TM^ Matrigel^TM^ Matrix | | | | Fischer Scientific | | CB-40234 | |
| VivoGlo^TM^ Luciferin | | | | Promega Life Sciences | | P1041 | |

| **Transcript** | **Forward primer** | **Reverse primer** |
| --- | --- | --- |
| Actin | CAACTGGGACGACATGGA | GTTGGCCTTGGGGTTCAG |
| TBP | TGCACAGGAGCCAAGAGTGAA | CACATCACAGCTCCCCACCA |
| Caveolin-1 | ACCCACTCTTTGAAGCTGTTG | GAACTTGAAATTGGCACCAGG |
| β-Catenin | TGTGGATACCTCCCAAGTCC | TCATTGCATACTGTCCATCAA |
| CD44s | CTGTACACCCCATCCCAGAC | TGTGTCTTGGTCTCTGGTAGC |
| E-Cadherin | ATGCTGAGGATGATTGAGGTGGGT | CAAATGTGTTCAGCTCAGCCAGCA |
| MMP-9 | CTGCCAGGACCGCTTCTACT | CTCAGGGCACTGCAGGATGT |
| CYP3A4 | TGGTGGACTCGCCTGTAATC | GGTGCAATCTCAGCTCACTTC |
| RHAMM | CAGCTGGAAGATGAAGAAGGA | GCATCTAGTTGTAGCTGAAAAGG |
| Snail | GAGGCGGTGGCAGACTAG | GACACATCGGTCAGACCAG |
| A9 | ACATCATGCACTTGGAGGAAC | CCGTAACAGGTGTTTGGAGAA |
| 18S | AGGATGAGGTGGAACGTGTG | GGCTAGGACCTGGCTGTATTT |
|  | | |
| **A9 ORF Clone** | **Supplier** | **Catalog** |
| UGT(UGT1A9) (NM-021027) Human Tagged ORF | OriGene Technologies | RC208861 |
| **shRNA** | **Supplier/Catalog** | **Sequences** |
| A9 | OriGene Technoogies/TL300662C (A9 shRNA#1) | CCATGCTCAATGGAAAGCACAAGTACGAA |
|  | OriGene Technologies/TL300662D (A9 shRNA#2) | CACTTGGAGGAACATTTATTATGCCACCG |
